# Finding the middle way: rethinking cGMP for sterility testing of cellular therapy products in minimal manipulation settings

**DOI:** 10.1128/jcm.01358-25

**Published:** 2025-11-13

**Authors:** A. J. Fenwick, A. Misra, I. Martin

**Affiliations:** 1 Section of Transfusion Medicine, Division of Laboratory Medicine, Cleveland Clinic Foundation 583189 , Cleveland, Ohio, USA; 2 Section of Microbiology, Division of Laboratory Medicine, Cleveland Clinic Foundation 583189 , Cleveland, Ohio, USA; 3 Department of Pathology and Laboratory Medicine, Dartmouth Health 4456, Lebanon, New Hampshire, USA; Vanderbilt University Medical Center, Nashville, Tennessee, USA

**Keywords:** value, quality, sterility, bone marrow, stem cells, cellular therapy, cGMP

## LETTER

The recent discussion on equipment validation for sterility testing (1) describes the intensive processes involved in implementing current Good Manufacturing Practices (cGMP) in the clinical laboratory in the context of sterility testing for cellular therapy products. We believe that these, and prior, expert guidance for sterility testing (2, 3) should more explicitly advocate for a nuanced, risk-based approach that provides clarity for minimal-manipulation, hospital-based cellular therapy laboratories (CTLs) whose primary function is direct clinical transplant support rather than high-manipulation manufacturing of cellular therapy products.

In high-volume research or commercial product manufacturing, maximalist (cGMP) sterility testing approaches are appropriate and necessary, considering the extensive product manipulation, large volume of products handled, and complex processing. These frameworks emphasize full cGMP-level aseptic controls, frequent requalification, and extensive environmental monitoring to ensure batch consistency and patient safety across distributed therapies. In contrast, hospital-based CTLs operate in a fundamentally different context, handling minimally or non-manipulated products in small volumes (i.e., numbers of products processed) with rapid turnaround to patient infusion. Many of these products comfortably fall under the regulatory framework of Section 361 and current Good Tissue Practices (cGTP). As Gebo et al. (1) state, Section 361 remains a regulatory gray area, but definitionally these products should not be exposed to the same cGMP regulations. Indeed, applying cGMP in the context of sterility testing for a hospital-based CTL and its associated clinical microbiology laboratory is disproportionately and prohibitively burdensome, unsustainable, and risks diverting resources away from direct patient care activities without proportionate benefit. There is a strong precedent for trusting the clinical quality systems implemented within a clinical microbiology laboratory to manage microbial testing and guide assessment in platelet sterility testing for hospital-based blood donor centers or high-risk solid organ transplants without falling under FDA manufacturing oversight. This mirrors the role of sterility testing in hospital-based CTLs and is a part of the clinical care continuum.

Additionally, many of the cGMP principles—sterility testing, rigorous environmental monitoring, and clean/aseptic technique—are already integrated into laboratory operations through cGTP requirements and accreditation standards (4), though with a far more risk-based and clinically aligned approach. Suggesting that cGMP requirements are best practice in this context creates unnecessary hurdles for clinical laboratories to provide in-house testing. Furthermore, the broader diagnostic stewardship literature (5–7) demonstrates that indiscriminate testing can lead to low-value results, increased risk of false-positive results, overuse of material and manpower resources, and opportunity costs. Stewardship principles emphasize aligning test scope and frequency to the clinical context, pretest probability, and organizational capacity to maximize health care value (where value = benefit/cost).

The recent literature on separate fungal cultures for CTL sterility testing is a prime example of how excessive testing can become counterproductive—undermining value-based risk assessment and resulting in unintended harmful consequences (2). Most hematopoietic stem cell products are now collected peripherally in a closed sterile system via apheresis. Whether performing apheresis collections on allogeneic (healthy) or autologous donors, the risk for environmental fungal contamination is extremely low. In contrast, bone marrow collections/harvests have a higher risk of environmental contamination, since the collection system cannot be completely closed off. After performing risk assessment utilizing historic data and the principles of diagnostic stewardship, the laboratory of one author added *targeted* additional fungal sterility cultures for bone marrow harvest products only. The basis for this decision was that, while not zero, the risk of apheresis product fungal contamination was significantly outweighed by the risks and consequences of higher rates of false-positive sterility cultures in these patients. This was achievable only through a collaborative relationship with the in-house clinical microbiology laboratory to better understand the limitations of current testing, collaborating in the validation design and benefiting from the rapid turnaround time and expert interpretation. Performing sterility testing in-house allows for the assessment of potential contamination in context, with nimble initiation of additional investigations, engagement with infection control and the patient-facing bone marrow transplant team, and ultimately, response in real time. This partnership enables a focused, high-quality, risk-based approach rather than outsourcing to an external cGMP laboratory, which would result in longer testing turnaround times, increased costs, and application of overly rigid binary standards for testing that do not reflect clinical realities.

We advocate for nuanced, risk-based expert guidance that clearly delineates the differences between *requirements* for manufacturing and minimal-manipulation environments (8). For hospital-based CTLs, consideration of new processes should be grounded in the principles of cGTP and diagnostic stewardship, ensuring high-quality and high-value care and patient safety, while avoiding low-value recommendations. Such an approach aligns with the goals of real-world medical practice—preserving patient safety and maintaining regulatory compliance while supporting timely access to life-saving therapies.
